# Manifesting Ecologic and Microbial Connections

**DOI:** 10.3201/eid1204.AC1204

**Published:** 2006-04

**Authors:** Polyxeni Potter

**Affiliations:** * Centers for Disease Control and Prevention, Atlanta, Georgia, USA

**Keywords:** Art and science, emerging infectious diseases, alexis rockman, manifest destiny, Brooklyn bridge, new york

**Figure:**
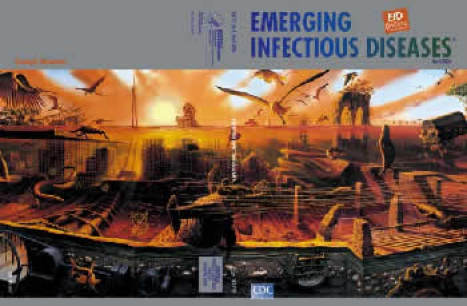
Alexis Rockman (b. 1962). Manifest Destiny (2003–2004) (detail). Oil and acrylic on 4 wood panels (2.44 m × 7.32 m). Brooklyn Museum, New York. Courtesy of the artist.

"I'm a pop artist using natural history as my iconography," Alexis Rockman has said of himself (*1*). Pop art, a movement that coincided with the youth and music phenomena of the 1950s and 1960s, draws its subject matter from the modern urban consumer experience, adopting popular culture icons and introducing them to the art world, much as American artist Andy Warhol incorporated and immortalized in his work canned soup and actress Marilyn Monroe (*2*). A man of his times, Rockman lives and breathes the culture of his native New York City, drawing inspiration from it, absorbing its trends, obsessions, conflicts, and fears, which he then brings to life in fantastic images created from nature.

Apart from a brief stay in a remote area of Peru, where his mother, anthropologist Diana Wall, did field work, Rockman grew up an urban child in an Upper East Side apartment oddly populated with newts, cats, boa constrictors, iguanas, tortoises, and lizards. He collected specimens and kept turtles and poisonous dart frogs, which he drew from a very young age, and planned to be a scientist. His playground was the American Museum of Natural History, where his mother worked with anthropologist Margaret Mead. The museum's extensive collections and dioramas became an influential part of his childhood. The artists who created the dioramas, he later said, were guided by the same painters who inspired him, Thomas Cole, Frederic Church, Albert Bierstadt (*3*).

His interest in zoology and botany was rivaled by other interests, among them film making and animation. He studied at the Rhode Island School of Design and graduated from New York's School of Visual Arts with a major in illustration. He worked as columnist and illustrator for Natural History magazine, while he gradually moved toward fine arts and started to show his work in solo and group exhibitions. A major influence was artist Ross Bleckner, whom he served as assistant for a time and who advised him to move toward modernism (*4*).

A leader among contemporary artists returning to figurative content, Rockman wants to paint what he sees (*5*). Taken with the natural world, he studies not only nature's creatures but also the puzzles surrounding them: their origins, survival, adaptability, evolution. Plants and animals are photographed and researched in libraries, their native habitats, or the Bronx Zoo. He delves into taxonomy and molecular biology and has enlisted the help and gained the following of paleontologists, biologists, ecologists, ichthyologists, and other scientists, who provide him clues to the accuracy of his exacting images. He has traveled to the rainforests of Brazil and Guyana in search of authentic specimens and to the South Pacific to sketch the extinct Tasmanian tiger in a local laboratory (*6*). He counts Charles Darwin as a mentor.

A combination of natural science and fantasy, his work explores the predatory relationship between nature and culture. Inspired equally by scientific curiosity and artistic compulsion, his startling images are at once literal, naturalistic, and entirely imaginary. Challenging the way we see and categorize the world, he questions human-animal-nature interaction by creating "in your face" scenarios based on vital popular culture dilemmas, among them genetic engineering and global warming.

"He tweaks my cerebrum," late professor Stephen Jay Gould said of Rockman (*7*). His snakes grow legs and chickens sport multiple sets of wings. Kangaroo-sized rats stroll across futuristic landscapes. A pig harbors human organs for harvest, and grossly oversized parasites, ticks, ants, and viruses populate his large surreal scenes. Botanical compositions, swarming with nature's less appreciated creatures and extinct or mutant forms feature aquatic or tree-sized dandelions. Humans are rarely present, though human handiwork always is. Riddles and humor are mixed in with actual soil, mud, sand, vegetation, and other collage materials, adding tactile interest to rich layers of color and varnish, which create a highly finished, luminous effect.

For nearly two decades, Rockman has worked from his studio in TriBeCa (Triangle Below Canal, between the Hudson River and Broadway), transforming historical culture into naturalistic images. Referring to himself as a "paleogeek," he favors large prehistoric landscapes reinterpreting the ecologic past and still lifes exploring the evolutionary future.

In Manifest Destiny, on this month's cover, Rockman imagines Brooklyn 3,000 years in the future. Fueled by exhaustive research, his artistic imagination produces a panoramic view of Brooklyn Bridge and environs. The polar ice caps have melted and the borough is under water. An eerie orange glow permeates layers of underwater ruins covered with slime and inhabited by weird creatures. In what the painter has referred to as "democratic space," tropical migrants paddle with newfangled mutants and everyday pests.

"I'm dying to see what scientists will think," Rockman said, while still working on the painting, transforming technical information from his research into visual language (*3*). In this restructured environment, geology is turned on its ear, along with the food chain. Snakehead fish swim by a giant red blood cell infected with HIV. Jellyfish tentacles stretch halfway across the seascape, past a two-tailed salmon. Flocks of wild birds hover above the waterline. Minute life forms (*Pfisteria*, adenovirus, SARS-associated coronavirus, West Nile virus), enlarged against the ruins, signal the survival of the unexpected. A galleon rests near the wreckage of a nuclear submarine. And the grand bridge lies broken, a fossil amidst decaying structures and vegetation.

Rockman's haunting vision of the future is rich with cultural and evolutionary undertones. The geologic, botanical, and zoologic clues to the future, rooted in the past and buried in the lurid reds of rust and pollution, are well understood by scientists. For ecologic disaster and disease emergence evolve along the same path, guided by the same factors: human demographics and behavior, technology and industry, economic development and land use, international travel and commerce, microbial adaptation and change, and the breakdown of public health measures (*8*).

And while short-term risk for epidemics after geophysical disasters may be low (*9*), long-term effects of ecologic change on disease emergence, aptly shown in the exaggerated size of viruses (e.g., HIV), are huge. Rockman's meticulously drawn mutants, alluding to genetic engineering or environmental pollution, also articulate dilemmas inherent in disease control: because of microbes' evolutionary potential, our very drugs or pesticides may contribute to selection of mutations, adaptations, and migrations that enable pathogens to proliferate and nonpathogens to become virulent. Manifest or not, the destiny of humans, animals, and the natural environment is inextricably interlinked.
